# Multiplane 2.5D microscopy for high-throughput high-resolution tissue imaging

**DOI:** 10.1117/1.JBO.30.10.106502

**Published:** 2025-10-17

**Authors:** Le-Mei Wang, Dhruvam Pandey, Wencai Zhang, Kyu Young Han

**Affiliations:** a University of Central Florida, CREOL, The College of Optics and Photonics, Orlando, Florida, United States; b University of Central Florida, College of Medicine, Burnett School of Biomedical Sciences, Orlando, Florida, United States

**Keywords:** extended depth of field, 2.5D imaging, multiplane, high-throughput, tissue imaging

## Abstract

**Significance:**

Fast, high-throughput fluorescence imaging is essential for numerous biomedical applications, particularly in high-resolution volumetric tissue analysis.

**Aim:**

We aim to develop an imaging strategy that combines the strengths of multiplane microscopy and extended depth-of-field (EDOF) microscopy and to characterize its performance on tissue samples.

**Approach:**

We employed 2.5D microscopy, an EDOF approach optimized for high-resolution imaging, and integrated it with a quad-plane image splitter. This technique enables simultaneous capture of four focal volumes using a single camera, allowing volumetric imaging of ∼16 to 20  μm thick mouse and human tissues prepared as frozen or formalin-fixed, paraffin-embedded sections.

**Results:**

Our approach achieves a 25-fold reduction in image acquisition time compared with conventional z-scanning widefield microscopy. For example, a 2  mm×2  mm×16  μm volume can be imaged in 4.7 min, down from ∼2  h. We further demonstrate compatibility with multicolor imaging and successful application to nucleus segmentation for downstream analysis.

**Conclusions:**

This imaging technique provides a promising tool for tissue analysis, offering significant improvements in volumetric imaging speed with minimal compromise in spatial resolution and sensitivity.

## 1 Introduction

Histological analysis of biological tissues has long been a fundamental approach for studying cellular characteristics, identifying pathological changes, and monitoring human health.^1^^,^^2^ Despite advancements, the demand for fast, sensitive, high-resolution volumetric imaging remains crucial.^3^ Common techniques such as widefield imaging,^4^^,^^5^ confocal microscopy,^6^^,^^7^ and light-sheet microscopy^8^^,^^9^ are widely used; however, they typically require axial scanning of a bulky stage to sequentially capture information from each plane, which is time-consuming and limits throughput.^10^^,^^11^

To address these challenges, several strategies have been proposed for fast volumetric imaging,^12^ which primarily eliminate the need for serial z-scanning. For instance, multifocal or multiplane microscopy captures fluorescence signals from different depths simultaneously using diffractive optical elements,^13^^–^^16^ image-splitting optics^17^^–^^21^ or a microlens array^22^^–^^24^ on single or dual camera sensors. Although these methods offer high-speed volumetric imaging, they are often constrained by inherently low signal-to-noise ratios and a reduced field of view (FOV) as the number of imaged planes increases. When depth-resolved information is less critical, extended depth-of-field (EDOF) imaging serves as an efficient alternative for fast volumetric imaging.^25^^–^^30^ This approach projects volumetric information onto a 2D image plane by manipulating emitted fluorescence light at the conjugate back focal plane (cBFP) of an objective lens.^31^^,^^32^ Recently, 2.5D microscopy (2.5DM), a highly optimized form of EDOF imaging designed for high-resolution studies and single-molecule applications, has shown promising results.^33^^–^^35^ It has demonstrated a 10-fold improvement in throughput for immunofluorescence and single-molecule RNA imaging of mammalian cells^33^ as well as extended observation in single-particle tracking.^35^ Nevertheless, as the depth of field of 2.5DM is limited to only 4 to 5  μm with a high numerical aperture (NA) objective, it is inevitable to obtain multiple 2.5D images at different depths for thick samples.^34^

Here, we introduce a fast volumetric imaging technique that integrates the merits of multiplane detection and 2.5DM. By implementing a quad-plane image splitter on a single camera, we expand the depth coverage from 4 to 16  μm. In addition, each focal volume captures projected signals over a 4  μm thickness, with each plane providing coarse depth information while maintaining high spatial resolution. We demonstrate the benefits of multiplane 2.5D microscopy through high-throughput tissue imaging over a $2\times 2\times 0.016\;{\mathrm{mm}}^{3}$ volume in under 5 min—achieving a 25-fold increase in throughput compared with conventional widefield microscopy. Our approach is validated in multicolor fluorescence imaging and various sample types, including frozen and formalin-fixed, paraffin-embedded (FFPE) tissues.

## 2 Materials and Methods

### 2.1 Optical Setup

**Fig. 1:**
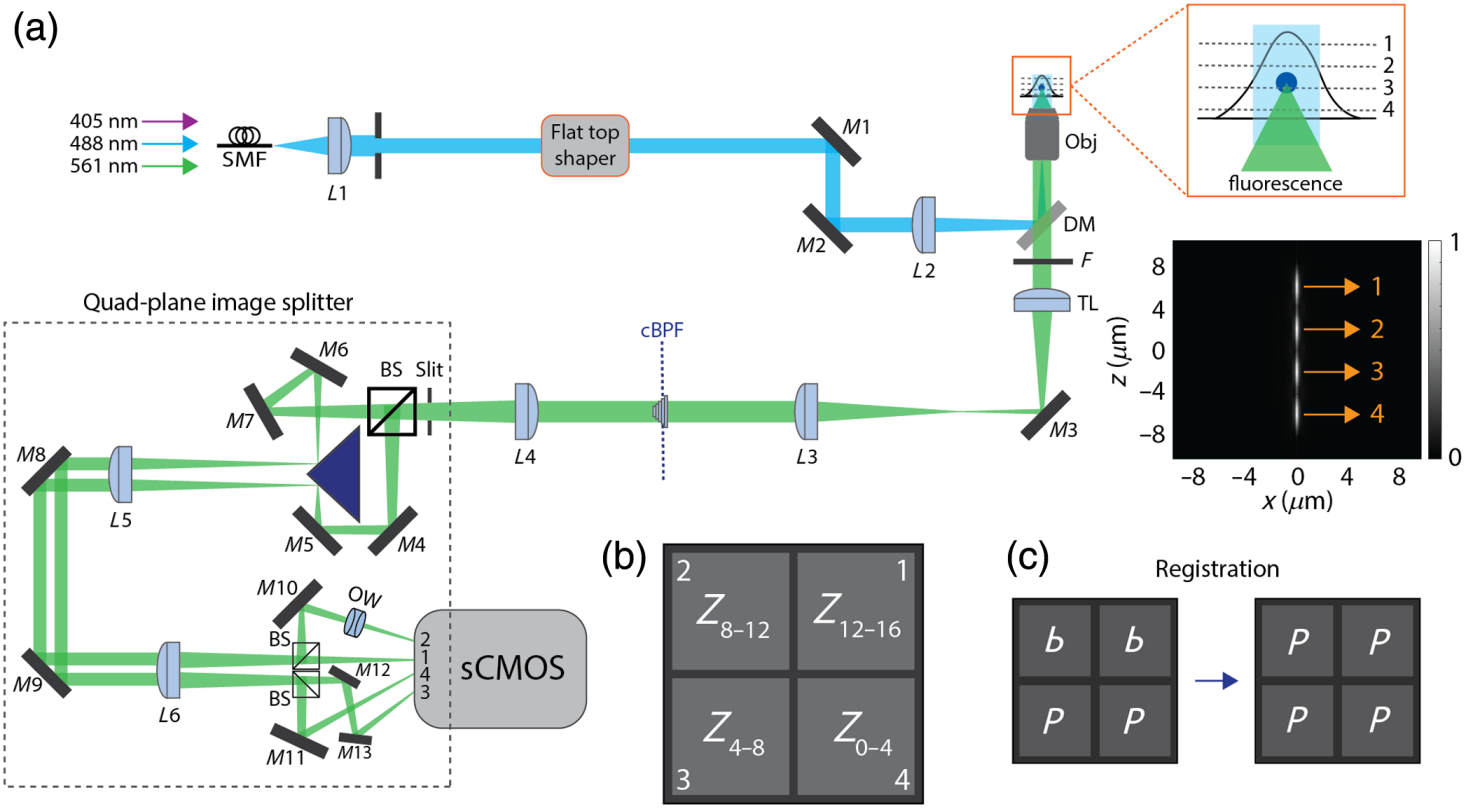
Multiplane 2.5D microscope. (a) Optical setup. BS, 50:50 beam splitter; DM, dichroic mirror; F, filter; L1−6, lenses; M1 to M13, mirrors; Obj, objective lens; OW, optical window; SMF, single-mode fiber; TL, tube lens. cBFP denotes conjugate back focal plane. (Right) Simulated PSF along the z-axis. Each PSF is extended to a 4  μm in depth and evenly distributed at different focal planes. (b) Four focal volumes recorded on the sCMOS camera. (c) Registration scheme of measured images. A letter P is displayed on each sub-image to help with visualization.

All images were captured by our custom-made 2.5D microscope^35^ with an addition of a quad-plane image splitter [Fig. 1(a)]. Three lasers (405, 488, and 561 nm; Cobolt, Solna, Sweden) were coupled into a single-mode fiber (P5-488PM-FC-2), and their output powers were controlled by polarizing beam splitters and half-wave plates. The fiber output was collimated with a lens (L1, f=100  mm) followed by a flat top shaper (TSM25-10-S-B; Asphericon, Jena, Germany) to generate a uniformly distributed excitation beam^36^ and then focused onto the back focal plane of an objective lens (Olympus UPlanSApo 60×/NA1.2, water immersion) through L2 (f=300  mm) and reflected by a multiband dichroic beam splitter (TRF89901; Chroma, Bellows Falls, VT, United States). The illumination beam was slightly inclined to reduce the unwanted out-of-focus background. The fluorescence signal was captured by the same objective, passed through a multibandpass filter (ZET405/488/561/640; Chroma). An intermediate image generated by a tube lens (f=180  mm) was relayed by two 1:1 image system (L3 and L4, f=250  mm; L5 and L6, f=500  mm) and captured by a scientific complementary metal oxide semiconductor (sCMOS) camera (Zyla 4.2 PLUS; Andor, Belfast, United Kingdom). A total magnification of our imaging system was 60, corresponding to ∼108  nm/pixel. All the optical components were purchased from Thorlabs (Newton, New Jersey, United States), unless specified.

To image four focal volumes on a single camera, the fluorescence signal was first split into two paths by a 50:50 beam splitter (BS013). Auxiliary mirrors (M4 to M7) and a right-angle prism mirror (MRAK25-P01) allowed the split beams to travel in parallel with the same optical path lengths. We used the beam diameter passing through the lens L5 during alignment as a reference. Because the two beams must have the same size, we placed an iris with the same diameter to ensure equal path lengths. Each beam was split again with another 50:50 beam splitter (BS004) before entering a camera. To achieve a 4  μm separation between each focal volume (Δz=4  μm), optical path lengths were adjusted accordingly. Specifically, using fluorescent beads immobilized on a coverslip, we first obtained an in-focus image on the first quad-plane ($Z_{12-16}$). The sample was then axially shifted by 4  μm using a piezo z-stage, and the mirrors were adjusted until the second quad-plane ($Z_{8-12}$) yielded an in-focus image. This procedure was repeated for the third and fourth planes. Folding mirrors (M10 to M13) ensured generating different optical path lengths for each focal plane, resulting in a set of four images on the camera. For the focal volume 2, due to the physical constraint, multiple windows (WG10530-A) with a total thickness of 18 mm were inserted to effectively shorten its optical path length. An adjustable slit (VA100CP) was inserted to generate a clear boundary of each image on the camera. Each quadrant image with ∼870×750  pixels ($∼94\times 81\;{\mu m}^{2}$) represents a focal volume spanning a 4  μm imaging depth at z=2, 6, 10, and 14  μm, denoted as $Z_{0-4}$, $Z_{4-8}$, $Z_{8-12}$, and $Z_{12-16}$, respectively [Fig. 1(b)]. For extended depth of field imaging, a layer cake (OP-12301-400-700, LightMachinery, Ottawa, Canada) was used instead of a phase-only spatial light modulator^33^^,^^34^ due to its ease of use and high transmission efficiency.^35^ Positioned at the conjugate back focal plane of the objective, the layer cake generates an axially elongated point spread function (PSF) as an incoherent superposition of multiple annular apertures.^37^ Although the multi-layered glass consists of five layers, we utilized only four to achieve the desired extended depth of field.

A sample was moved using a motorized XY-stage (SCAN IM 120×80; Marzhauser, Wetzlar, Germany) and a piezo z-stage (Z-INSERT; PiezoConcept, Bron, France). MicroManager^38^ was used to capture all image sequences, including the z-stacks and high-throughput grid images. For fast high-throughput imaging, a microcontroller module (Mega 2560 Rev3; Arduino) was integrated into MicroManager software to receive firing signals from the camera and generate output signals for digitally switching lasers on and off via TTL signals. This allowed acquiring multicolor images at each position before moving to the next grid.

### 2.2 Image Processing, Analysis, and Simulation

For accurate image analysis, we cropped the captured images into four sub-images and vertically flipped $Z_{8-12}$ and $Z_{12-16}$ relative to $Z_{0-4}$ and $Z_{4-8}$ due to the reflection caused by an odd number of mirrors in the detection path. Using $Z_{12-16}$ as a reference, we performed registration and alignment of the other focal planes with z-stacks of 2D bead samples [Fig. 1(c)]. The results confirmed that each bead was well aligned across all the planes. The large FOV tissue images were stitched using the FIJI grid/collection stitching algorithm, where a regression threshold of 0.3, a max/average displacement threshold of 2.5, and an absolute displacement threshold of 8.0 were used.

We employed the Cellpose framework, a widely used deep learning–based model for generalized segmentation tasks, to perform nucleus segmentation.^39^ Before segmentation, the input images underwent preprocessing to enhance their quality and improve segmentation accuracy. Initially, bilateral filtering^40^ was applied to suppress noise while preserving edge details, ensuring that fine structural information within the nuclei was retained. After preprocessing, we utilized the Cellpose model with the model type parameter set to “nuclei,” enabling the framework to accurately segment nuclei based on its pretrained feature representations. To predict and verify our imaging system, we simulated the detection PSF at four individual planes using Fourier optics in MATLAB as we did previously.^35^

### 2.3 Sample Preparation

For the 2D bead samples, 100 nm yellow–green fluorescent beads (F8803; ThermoFisher, Waltham, Massachusetts, United States) or 100 nm TetraSpeck Microspheres beads (T7279; ThermoFisher) were immobilized on a coverslip coated with poly-L-lysine (P8920; Sigma, St. Louis, Missouri, United States). The coverslip was sealed with a glass slide using water as the mounting medium. For 3D hydrogel samples, the hydrogel was prepared as previously described.^35^ In brief, a 7.5% acrylamide: bisacrylamide (29:1) (National Diagnostics, Atlanta, Georgia) solution was mixed with 0.2% (v/v) tetramethylethylenediamine (TEMED; T7024, Sigma, Kawasaki, Japan) and 0.02% (w/v) ammonium persulfate (A3678; Sigma) in 0.5× TAE buffer. Yellow–green fluorescent beads were introduced into the hydrogel solution at a final concentration of 5% (v/v). After thoroughly mixing with a vortex mixer, the solution was rapidly injected into a flow chamber. After 10 min, the solution transformed into a gel, and images of the 3D hydrogel samples were captured.

Human normal lung FFPE sections (VitroVivo Biotech, Rockville, Maryland, United States) with a thickness of 20  μm were placed on glass slides and baked at 60°C for 30 min. Subsequently, the sections underwent deparaffinization and hydration through sequential washes in xylene and a graded ethanol series to water. Following three washes with PBS, tissue sections were incubated in the dark at room temperature with 5  μg/mL acridine orange (A1301; ThermoFisher) for 15 min for nuclear staining. After a brief rinse with PBS, the tissue sections were mounted using ProLong Diamond Antifade Mountant (P36965; ThermoFisher) and covered with a coverslip. In addition, a frozen mouse kidney sample (16  μm thick) labeled with DAPI, Alexa Fluor 488 wheat germ agglutinin (AF488), and Alexa Fluor 568 phalloidin (AF568) was purchased from ThermoFisher (F24630).

## 3 Results

### 3.1 Characterization of Quad-Plane 2.5D Microscope

**Fig. 2:**
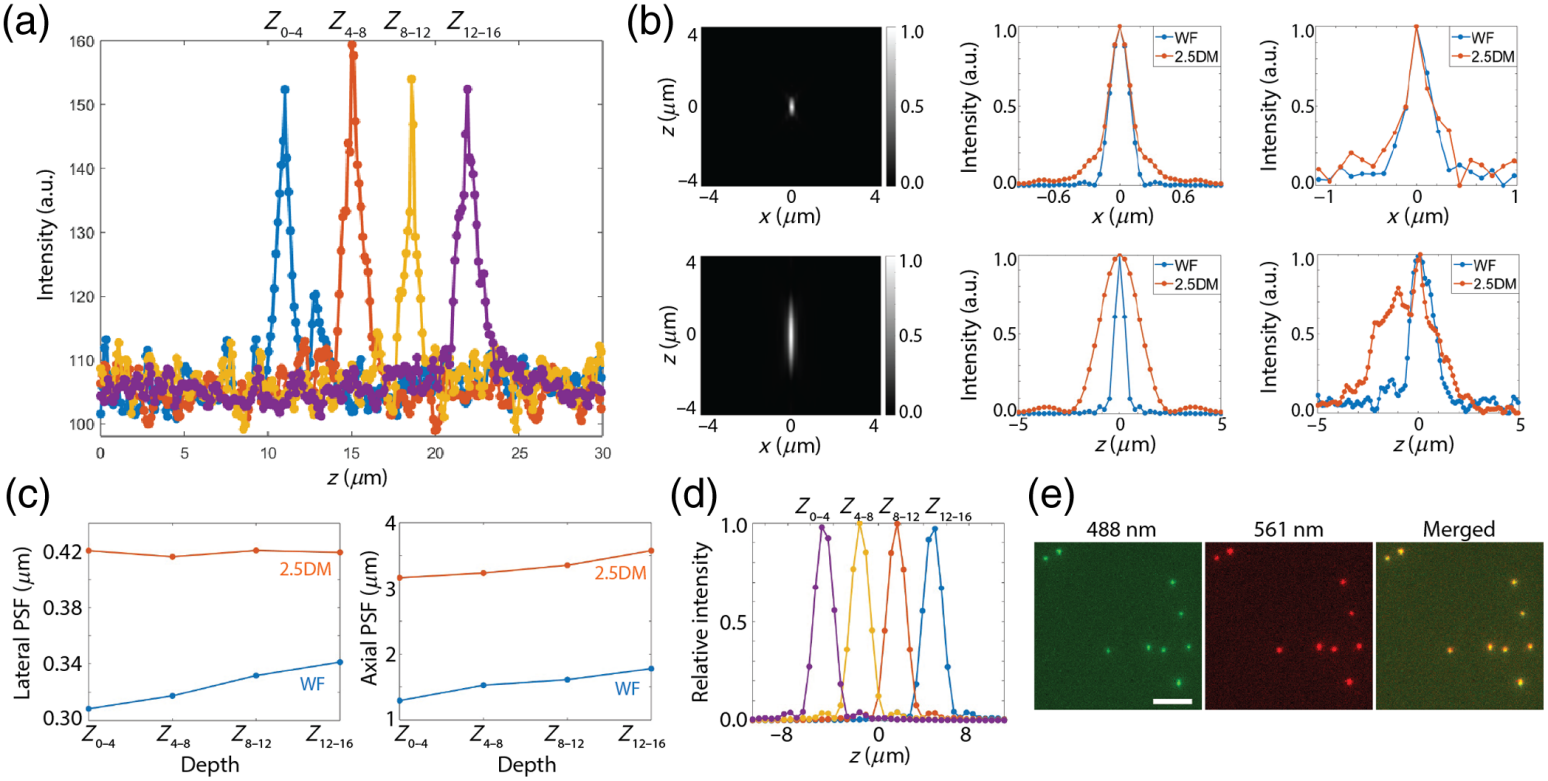
Characterization of multiplane 2.5D microscopy. (a) Experimental measurement of inter-plane distances without the cake layer. (b) Simulated PSFs (left), simulated (middle), and experimental (right) line profiles of fluorescent beads for widefield microscopy and 2.5DM. (c) Measured lateral and axial PSFs at four focal planes. (d) Simulated axial profiles for each focal plane. (e) Two-color images of fluorescent beads. Scale bar, 5  μm.

We first measured the inter-plane distance of a quad-plane image splitter before inserting the layer cake. We imaged 100 nm fluorescent beads (505/515) immobilized on a coverslip using a 488 nm laser by axially scanning the sample. Beads sequentially detected in each focal plane were selected, and their intensity distributions along the z-axis were analyzed. As expected, peak intensities were observed at ∼4  μm increments [Fig. 2(a)], confirming that the four focal planes were evenly spaced as designed. Each peak intensity exhibited small variations (<15%), indicating that all optical paths had comparable transmission efficiency and that depth-induced aberrations were negligible.

Next, we inserted the layer cake and captured four focal volume images, $Z_{0-4}$, $Z_{4-8}$, $Z_{8-12}$, and $Z_{12-16}$ for the fluorescent beads. We then measured the full width at half maximum (FWHM) of lateral and axial profiles from the resulting 2.5D images. The measured FWHMs were 420±10  nm and 3.89±0.22  μm, respectively, compared with 308±12  nm and 1.15±0.13  μm for widefield imaging [Fig. 2(b), right]. These indicate a ∼3.4-fold extension in the axial direction and a ∼1.35-fold broadening in the lateral direction. Our experimental results aligned well with simulation predictions, which showed a ∼3.7-fold axial extension [Fig. 2(b), left]. We measured the PSFs at various depths and found negligible variations in both lateral and axial dimensions across each focal volume [Fig. 2(c)], consistent with simulation results [Fig. 2(d)].

In addition, we imaged TetraSpeck beads to evaluate whether the layer cake and quad-plane image splitter introduce any wavelength-dependent lateral shifts. As shown in Fig. 2(e), the fluorescent beads excited by 488 and 561 nm light exhibited strong spatial overlap, indicating negligible chromatic aberration. As a result, no additional image registration was necessary.

### 3.2 Fast Volumetric Imaging

**Fig. 3:**
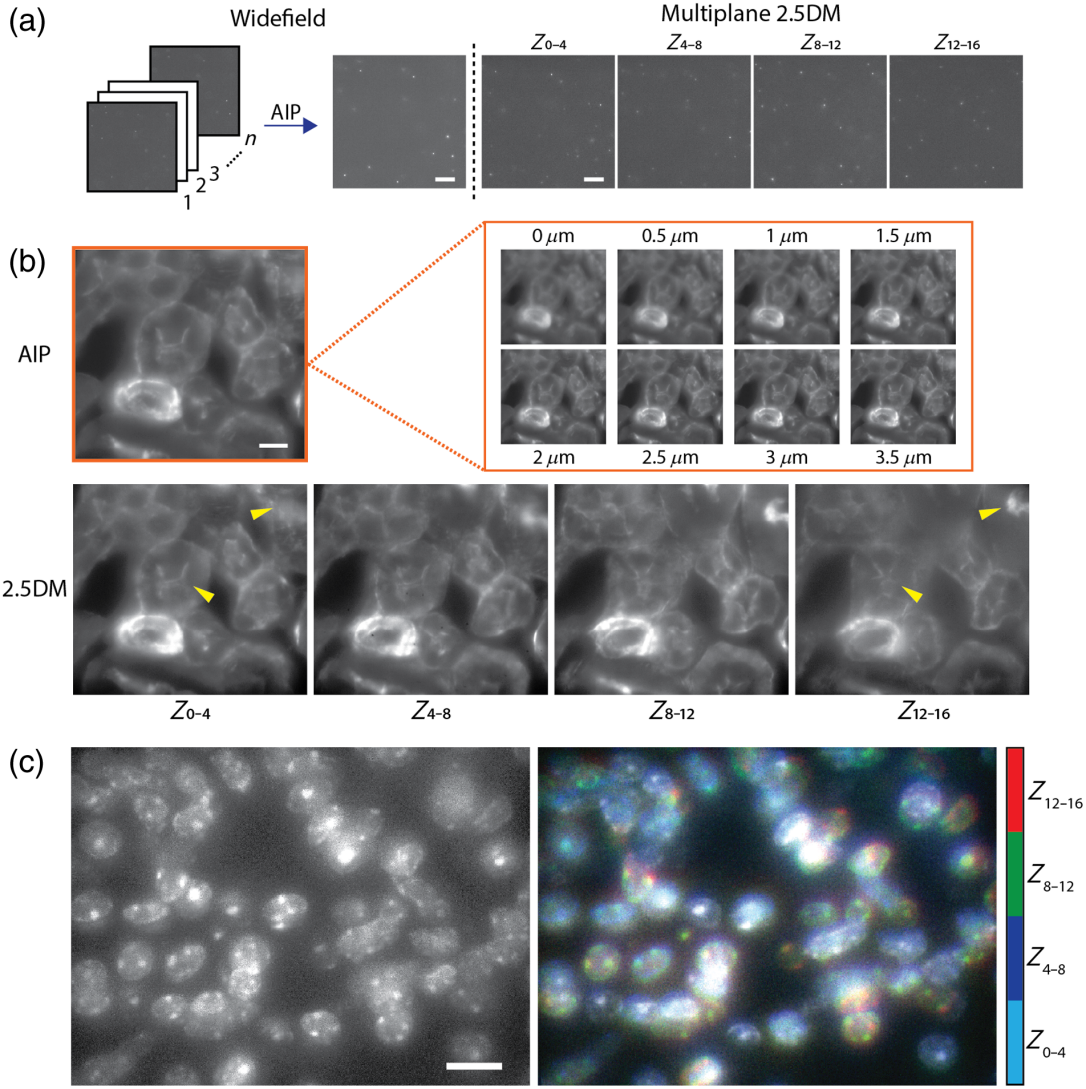
(a) Fluorescent beads embedded in 3D hydrogels imaged by widefield microscopy (left) and multiplane 2.5DM (right). An AIP image was generated by z-stack widefield images. (b) Frozen mouse kidney tissues (AF488 phalloidin) captured with widefield microscopy and multiplane 2.5DM. Examples of depth-dependent structural changes were displayed with yellow marks. (c) Multiplane 2.5D images of mouse kidney tissues (DAPI) displayed with maximum intensity projection (left) and color-coded depth-resolved representation (right). Scale bars, 10  μm.

In conventional widefield volumetric imaging, serial z-scanning is the most time-consuming step but essential for capturing volumetric information. When using a high NA (NA>1.0) objective, a step size of 0.25 to 0.5  μm is typically required to satisfy the Nyquist criterion. To evaluate the speed advantage of our approach, we first imaged fluorescent beads embedded in 3D hydrogels and compared the performance of our detection system with that of conventional widefield microscopy. Using our multiplane 2.5DM, we simultaneously captured four depth-resolved images, each covering a 4  μm depth range, for a total axial coverage of 16  μm. As a reference, we acquired 64 images through serial z-scanning and generated average intensity projections (AIPs) for every 16-image stack, each corresponding to a 4  μm depth. As shown in Fig. 3(a), the multiplane 2.5D images showed a good agreement with the resulting AIP images in terms of contrast, demonstrating that our method can effectively capture coarsely depth-resolved volumetric information in a single shot.

Next, we imaged a 16  μm thick cryo-sectioned mouse kidney tissue labeled with Alexa Fluor 488 conjugated wheat germ agglutinin using 488 nm laser excitation. We first acquired 32 widefield images at 0.5  μm z-intervals without the layer cake to cover the full tissue depth. Similar to our approach with the 3D hydrogel sample, these z-stack images were then projected onto a 2D image using AIP [Fig. 3(b), top]. In comparison, we captured multiplane 2.5D images at four depths simultaneously using our imaging system. Both methods were conducted using the same exposure time (20  ms/frame) and illumination intensity. The 2.5D images yielded comparable structural details to the AIP images [Fig. 3(b)], effectively capturing the features of interest within the tissue volume. Remarkably, the total acquisition time was reduced from ∼1  s to 20  ms, demonstrating a substantial improvement in imaging speed. Another feature of our imaging system is its ability to provide coarse depth-resolved information, unlike other approaches that simply extend the depth of field across the entire volume. To demonstrate this, we imaged the same sample using 405 nm laser excitation to visualize nuclei. As shown in Fig. 3(c), individual nuclei located at different depths were clearly distinguishable through distinct color coding.

### 3.3 Large FOV Tissue Imaging

**Fig. 4:**
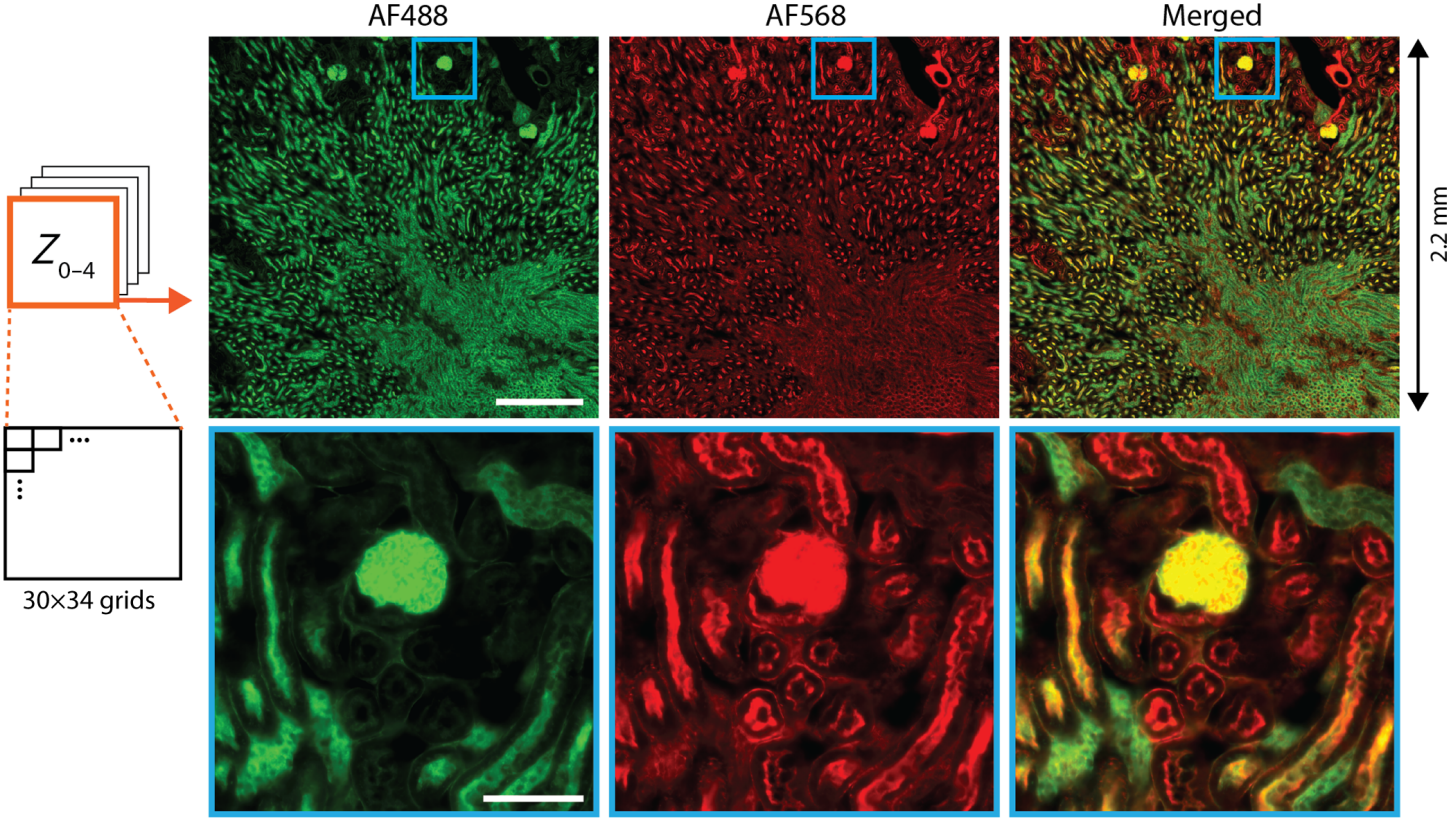
Two-color large FOV mouse kidney images (30×34 grids) recorded with multiplane 2.5DM. AF488 conjugated to wheat germ agglutinin (green) and AF568-phalloidin (red). Scale bars, 500  μm (top) and 100  μm (bottom).

We acquired large FOV volumetric images (2  mm×2  mm×16  μm) for a mouse kidney tissue using two color imaging (AF488 and AF568). To capture the entire sample efficiently, we employed the stop-and-snap strategy,^33^ where two-color images were rapidly acquired at each FOV while the stage was momentarily paused. A total of 1020 positions were imaged. For demonstration, we present one of the focal volumes, $Z_{0-4}$, from the quad-plane acquisition [Fig. 4]. After stitching the individual images, the two channels were merged into a composite image [Fig. 4, right]. We successfully imaged distinct spatial distributions of carbohydrates and actin structures. Remarkably, the total imaging time was reduced from ∼2  h to 4.7 min, highlighting the potential of our techniques for fast, high-throughput volumetric imaging of large tissue samples.

**Fig. 5:**
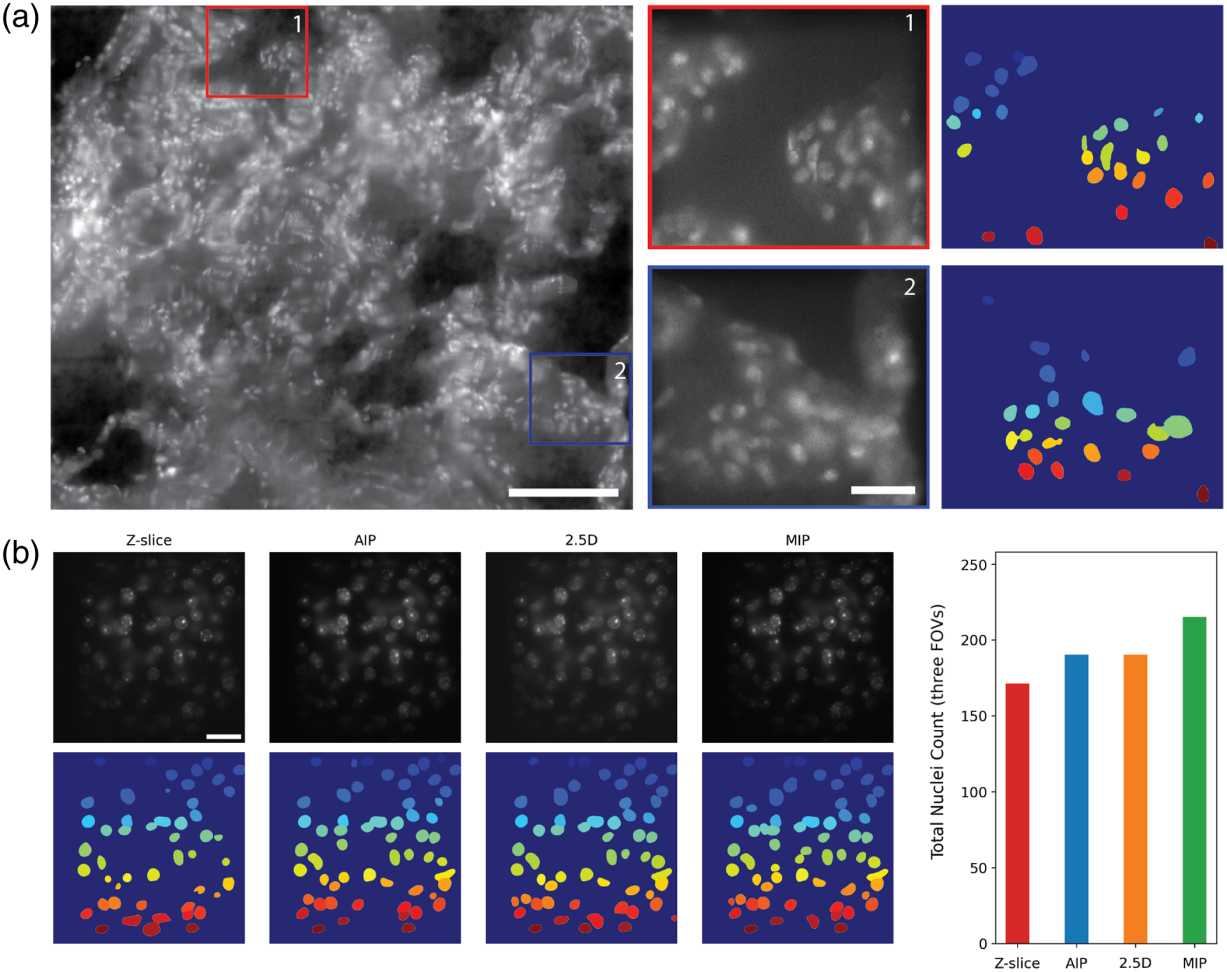
(a) Multiplane 2.5DM image of human lung FFPE tissue sections labeled with acridine orange (left) and nuclei segmentation results (right). Each nucleus is displayed with different colors. Scale bars, 100  μm (a, left) and 20  μm (a, middle). Each nucleus is assigned a distinct color using the jet colormap solely for visual clarity; the colors do not reflect any biological differences. (b) Nuclei segmentation in mouse kidney tissue using Cellpose across four images: three images correspond to a single central z-slice, an AIP, and an MIP of eight z-slices acquired with a conventional widefield microscope. The fourth image was acquired using 2.5DM. Top row: mouse kidney tissue images from different modalities; bottom row: corresponding segmentations. The bar graph (right) shows total nuclei counts across three FOVs. Scale bar: 20  μm.

Last, we evaluated whether our multiplane 2.5D images could be used for nucleus segmentation, a critical task for analyzing tissue organization, including both normal and aberrant architecture.^41^^,^^42^ We imaged a 20  μm thick human lung FFPE tissue ($∼0.5\times 0.5\;{\mathrm{mm}}^{2}$) labeled with acridine orange and performed nucleus segmentation using Cellpose.^39^ Despite elevated background levels, the majority of nuclei in the lung tissue were successfully segmented [Fig. 5(a)]. For quantitative comparison, we have further analyzed segmentation accuracy across four image types: (1) a single z-slice located at the center of the volume, (2) an AIP, (3) a maximum intensity projection (MIP) of eight z-slices acquired using a regular widefield microscope, and (4) a 2.5D image corresponding to the focal volume. As shown in Fig. 5(b), 2.5D imaging was able to segment 190 (88%) out of 215 nuclei in mouse kidney tissues, where MIP was used as the reference. These results indicate that multiplane 2.5D imaging can support reliable downstream analyses such as nucleus counting, morphology analysis, and nuclear heterogeneity.^43^ Note that we used raw images without deconvolution as inputs to nucleus segmentation.

## 4 Discussion and Conclusion

In this study, we introduced a high-throughput, fast volumetric imaging technique that significantly reduces acquisition time by combining multiplane imaging with EDOF imaging. Although widefield microscopy provides the highest spatial resolution with a relatively simple optical setup, it requires extensive z-scanning to capture volumetric information. Conventional EDOF imaging or multiplane imaging over a 16 to 20  μm axial range can alleviate this limitation; however, EDOF imaging suffers from high background signals and loss of depth information, whereas multiplane imaging has low detection efficiency and complex instrumentation. By contrast, our multiplane 2.5DM acquires volumetric data in a single shot by projecting fluorescence signals across a designed depth range, retaining the advantages of both techniques while minimizing their drawbacks. By integrating a quad-plane image splitter into the 2.5DM setup, we further extended the DOF and gained coarse depth-resolved information. Our multiplane 2.5DM demonstrated the ability to image large tissue sections up to 16  μm thick with high spatial resolution. Although our approach results in ∼1.35-fold broadening of the lateral PSF, it offers a clear advantage over low NA systems, which achieve similar axial extension (∼4-fold) but at the cost of >2-fold broadening in the lateral direction. Our imaging system is compatible with multicolor imaging and supports a variety of sample types, including frozen tissues and FFPE sections. Our approach offers advantages for high-throughput, rapid imaging, making it valuable for applications such as cancer tissue analysis, where large FOVs must be examined in a short time.^44^^,^^45^

Although the layer cake used in our setup cannot adaptively correct sample-induced aberrations and its axial PSF is less uniform than that of a well-designed phase mask,^34^ its high transmission efficiency and ease of integration make it well-suited for high-throughput imaging. Our quad-plane image splitter inherently yields ∼25% transmission efficiency per depth. Nevertheless, because tissue samples typically produce higher fluorescence signals than adherent cells, the impact of reduced detection efficiency on image contrast is minimal.

The total imaging depth can be controlled by increasing the number of focal planes, whereas the EDOF thickness for each plane can be tuned by modifying the number of layers in the layer cake. It is worth noting that alternative imaging approaches can be incorporated into multiplane EDOF imaging. For instance, Bessel beam^46^ or Bessel-droplet phase masks^47^ may provide greater extended imaging depth or reduced side lobes; however, they are generally more suitable for two-photon excitation applications. Although we used a single camera to capture four focal planes, resulting in a reduced FOV per plane, this limitation could be addressed using four separate cameras with the quad-image splitter. Such an approach will expand the FOV and further reduce acquisition time. In addition, background suppression through deconvolution^35^ may enhance the robustness of nucleus segmentation in multiplane 2.5D images. Given its speed, simplicity, and high efficiency, we anticipate that our approach will be a valuable tool for translational research.

## Data Availability

Data in this paper are available upon reasonable request to the corresponding author.
